# Compliance With Mobile Ecological Momentary Assessment Protocols in Children and Adolescents: A Systematic Review and Meta-Analysis

**DOI:** 10.2196/jmir.6641

**Published:** 2017-04-26

**Authors:** Cheng K Fred Wen, Stefan Schneider, Arthur A Stone, Donna Spruijt-Metz

**Affiliations:** ^1^ Department of Preventive Medicine University of Southern California Los Angeles, CA United States; ^2^ Center for Self-Report Science University of Southern California Los Angeles, CA United States; ^3^ Center for Economic and Social Research University of Southern California Los Angeles, CA United States; ^4^ Department of Psychology University of Southern California Los Angeles, CA United States; ^5^ mHealth Collaboratory University of Southern California Los Angeles, CA United States

**Keywords:** ecological momentary assessment, compliance, youth, mHealth

## Abstract

**Background:**

Mobile device-based ecological momentary assessment (mobile-EMA) is increasingly used to collect participants' data in real-time and in context. Although EMA offers methodological advantages, these advantages can be diminished by participant noncompliance. However, evidence on how well participants comply with mobile-EMA protocols and how study design factors associated with participant compliance is limited, especially in the youth literature.

**Objective:**

To systematically and meta-analytically examine youth’s compliance to mobile-EMA protocols and moderators of participant compliance in clinical and nonclinical settings.

**Methods:**

Studies using mobile devices to collect EMA data among youth (age ≤18 years old) were identified. A systematic review was conducted to describe the characteristics of mobile-EMA protocols and author-reported factors associated with compliance. Random effects meta-analyses were conducted to estimate the overall compliance across studies and to explore factors associated with differences in youths’ compliance.

**Results:**

This review included 42 unique studies that assessed behaviors, subjective experiences, and contextual information. Mobile phones were used as the primary mode of EMA data collection in 48% (20/42) of the reviewed studies. In total, 12% (5/42) of the studies used wearable devices in addition to the EMA data collection platforms. About half of the studies (62%, 24/42) recruited youth from nonclinical settings. Most (98%, 41/42) studies used a time-based sampling protocol. Among these studies, most (95%, 39/41) prompted youth 2-9 times daily, for a study length ranging from 2-42 days. Sampling frequency and study length did not differ between studies with participants from clinical versus nonclinical settings. Most (88%, 36/41) studies with a time-based sampling protocol defined compliance as the proportion of prompts to which participants responded. In these studies, the weighted average compliance rate was 78.3%. The average compliance rates were not different between studies with clinical (76.9%) and nonclinical (79.2%; *P*=.29) and studies that used only a mobile-EMA platform (77.4%) and mobile platform plus additional wearable devices (73.0%, *P*=.36). Among clinical studies, the mean compliance rate was significantly lower in studies that prompted participants 2-3 times (73.5%) or 4-5 times (66.9%) compared with studies with a higher sampling frequency (6+ times: 89.3%). Among nonclinical studies, a higher average compliance rate was observed in studies that prompted participants 2-3 times daily (91.7%) compared with those that prompted participants more frequently (4-5 times: 77.4%; 6+ times: 75.0%). The reported compliance rates did not differ by duration of EMA period among studies from either clinical or nonclinical settings.

**Conclusions:**

The compliance rate among mobile-EMA studies in youth is moderate but suboptimal. Study design may affect protocol compliance differently between clinical and nonclinical participants; including additional wearable devices did not affect participant compliance. A more consistent compliance-related result reporting practices can facilitate understanding and improvement of participant compliance with EMA data collection among youth.

## Introduction

### Background

There is a growing interest in studying the dynamic relationship among individuals’ experiences, social or physical environments, and behaviors. The assessment of these dynamic relationships is enhanced by the development of momentary data collection strategies, such as experience sampling methods (ESM) and ecological momentary assessment (EMA) [1]. Studies using these strategies usually require that their participants provide self-report ratings of their current or near-current experiences, environments, and behaviors. As summarized by Shiffman et al [1], these repeated “in the moment” measurements offer numerous methodological advantages over traditional assessment strategies. First, the momentary assessment of participants’ current or immediate past experiences or behaviors reduces the recall bias inherent in traditional retrospective survey methods. Second, the “in the moment” and “in the context” measurements collected in natural settings provide data that is more relevant to the current social or physical environments, thereby providing more ecologically valid data. Third, the daily intensive repeated measurements allow for examinations of immediate antecedents and consequences of behavior in real-time, capturing within-day, within-person behavior, and experience variations across time.

EMA studies can be broadly categorized into (1) time-based and (2) event-based designs. These strategies provide different insights about the study participants. The time-based strategy usually aims to acquire representative characteristics and patterns of behaviors and experiences across time, whereas a study using an event-based strategy aims to examine antecedents and consequences of specific behaviors or experiences [1]. On the basis of the study rationales, variations of time-based (eg, prompting participants at random times and within a window of time) and event-based (eg, participant self-initiated self-report in response to occurrence of specific events or prompted by sensed events, such as location via Global Positioning System (GPS), or bouts of physical activity via accelerometer) strategies have been used, either on their own or in combinations. Technology innovations have transformed and enhanced momentary data collections in natural settings during the past decade. Features specific to mobile technology have provided solutions for many challenges that early EMA researchers faced. For example, some noncompliant behaviors, such as backfilling, or completing all the assigned diaries in bulk at the same time [2], can be effectively addressed by disabling prompt access on a mobile-EMA platform after a specific time window. Furthermore, mobile technologies provide researchers with time-stamped data on participant compliance that allow for a more reliable and objective measurement of participant compliance as compared with traditional paper-and-pencil recall methods, which have been shown to produce an inflated compliance rate [3]. These noncompliant behaviors (eg, backfilling), as well as other sources of bias (eg, missing assessments due to engagement in other activities that compete for participants’ attention), can yield biased data that no longer corresponds to the moment when the behaviors or experiences of interest occurred, thereby reducing ecological validity of the collected data. A major strength of mobile technologies is the readily available features that can validate the timeliness of participants’ response (eg, built-in sensors, phone usage data, and automatic timestamp) that can objectively measure response compliance. These can enhance the validity of momentary data collected in mobile technology-based EMA studies. Various emerging mobile technologies have been incorporated into EMA studies throughout the past decade; for example, personal digital assistants (PDAs), palmtop computers, and more recently, smartphones. Among participants across all age groups, mobile device-based EMA studies offer promising opportunities for researchers to study behavior and experience, especially in the youth population. Youth are “digital natives” [4] and are considered adept in and comfortable with technology in their day-to-day life activities, for example, for communication [5,6], and for receiving health-related intervention materials [5]. The high acceptability and ubiquity of digital and mobile devices, along with the methodological advantages, presents valuable opportunities for researchers to engage young study participants in EMA studies. During the recent decade, mobile device-based EMA has been widely utilized to assess and understand the dynamic relationship among youth’s behaviors, experiences, and pertinent contextual information in the youth population.

Although collecting momentary data using mobile technologies offers many advantages, these advantages depend on the quality of the collected data. Incorporation of mobile technologies in EMA studies can facilitate momentary data collection with an improved measurement of compliance and possibly in a higher frequency than using more conventional collection techniques. Although this provides an opportunity to understand behavior on a more granular level, systemic missing data (eg, participant noncompliance or engagement in competing activities) still threatens data quality. As stated above, several features of mobile technologies can minimize the impacts of some types of noncompliance behavior (eg, backfilling) on data quality. Nonetheless, as EMA study protocols usually involve participants being repeatedly interrupted and asked to provide self-reported information, these demands on study participants can lead to high perceived participant burden, and to noncompliance [2]. In the context of mobile-EMA studies, possible sources of participant burden include, but are not limited to, the use of technology (eg, familiarity with the reporting platform and incorporation of additional wearable devices), technological issues (eg, problems with the reporting platforms), study design (length of monitoring and daily sampling frequency), and quality, complexity, and the duration of prompts. Nonetheless, there are a limited number of studies that systemically review participant compliance to EMA protocols [1,3,7-9] and only one review specifically focuses on youth populations [9]. In the review, Liao et al [9] included 13 studies and observed an average compliance rate of 71%. This review, although an important contribution to the literature, was limited to obesity-related behaviors and did not quantitatively examine relationship between participant compliance and study design factors. This systematic review and meta-analysis, therefore, expands upon Liao et al findings by examining compliance to EMA protocols in youth involving a variety of self-reported behaviors and experiences, and by quantitatively assessing the relationships between compliance and some aspects of study design. We restrict the inclusion to studies that use digital momentary assessment techniques where compliance is electronically time stamped on the momentary level.

### Aims of This Study

The first aim of this review is to describe the characteristics of EMA protocols conducted among pediatric populations across a wide spectrum of health behaviors. The second aim is to quantify overall compliance rates and to examine the association between study design factors (length of monitoring period and daily sampling frequency) and reported compliance using a meta-analytic approach. Studies using clinical and nonclinical samples were both included in the review; however, given that study populations and objectives often differ quite substantially for these types of studies, they were examined separately and the results were compared. The exploratory aim of this study is to examine the association between participant compliance and other pertinent study design variables (eg, inclusion of additional wearable devices and incentive structure) on a post hoc basis. Finally, this study will also provide recommendations for future research that incorporates mobile devices in collecting real-time self-reported data to maximize the advantages of EMA methodologies.

## Methods

### Data Acquisition

A comprehensive literature search was conducted using the publicly accessible academic literature search engines (PubMed, PsycINFO, *Journal of Medical Internet Research*, and Google Scholar) from inception to March 28, 2016. The search terms employed in this review were composed of two components: (1) terms related to EMA and (2) terms related to participants aged 18 years or younger. Terms related to EMA were “ecological momentary assessment,” “ecological momentary intervention,” “momentary,” “experience sampling methods,” “event sampling methods,” and “daily diary methods.” Terms related to participant age were “adolescent,” “child,” “children,” and “youth.” Additional empirical studies were identified from the citations of the articles.

### Inclusion and Exclusion Criteria

#### Criteria for Inclusion in the Qualitative Systemic Review

Abstracts and full articles of the retrieved titles were screened for relevance. The article selection strategies, inclusion criteria, and exclusion criteria were determined in consensus meetings among authors. In order to be included in the systematic review, studies were required to (1) be an empirical study; (2) employ EMA strategies, including diary methods with more than one entry per day, ESM, and event sampling methods; (3) utilize mobile technologies for EMA data collection (cell phones, PDAs, smartphones, and so on); and (4) include children or adolescent (age ≤18 years) participants. Studies that involved adult participants (age >18 years), in addition to children and adolescents, were only included in the review if separate analytic or descriptive results were presented for the children or adolescents subgroups. Studies with any of the following 5 exclusion criteria were excluded: (1) did not utilize any electronic, wearable, or mobile technology; (2) utilized paper-based diaries to collect momentary data; (3) collected momentary or diary data once or less than once per day during the monitoring period; (4) utilized call-based (phone interview) data collection; and (5) data collection did not take place in free-living natural settings.

#### Criteria for Inclusion in the Meta-Analysis

A subset of studies meeting the criteria for the systematic review was included in the meta-analysis portion of the study. To meet the criteria for meta-analysis, studies were required to report (1) sufficient information that permitted the calculation of an average compliance rate (eg, percentage of EMA prompts answered) to be used as effect size (ES), (2) number of participants in the study, and (3) daily prompting frequency and length of monitoring period.

### Meta-Analysis Procedures

**Figure 1:**
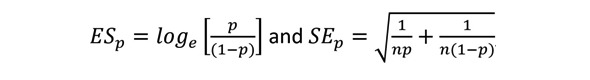
Calculation of effect size.

Random effects meta-analyses were conducted to (1) examine the average rate of compliance with EMA protocols pooled across all included studies and then across studies with clinical participants and nonclinical participants separately, and (2) to explore potential between *-* study differences in compliance rates based on daily prompting frequency and length of monitoring. A post hoc analysis was conducted to examine whether there is a difference in compliance rates among studies (1) with and without wearable devices *in addition to* the mobile-EMA platform and (2) with a fixed and incremental incentive strategy. In meta-analyses, study level averages are synthesized rather than individual participant data. Accordingly, compliance rates were operationalized as the average proportion of prompts completed by a participant in a given study (ie, the actual number of prompts completed divided by the number of prompts specified by the study protocol). To calculate an ES adequate for the analysis of proportions [10], the compliance rates were logit-transformed and standard errors were calculated accordingly as shown in Figure 1, where *p* is the proportion of completed prompts and *n* is the effective sample size in the study.

In this review, an adequate calculation of the standard errors (and hence the inverse variance weights used in meta-analysis) is complicated by the fact that EMA studies involve a nested study design, with multiple observations (prompts) clustered in participants. In this case, the effective sample size of each study needs to account for the clustered design. Following the methods recommended in the Cochrane Handbook for Systemic Review of Interventions [11], this can be achieved by adjusting the total sample size (ie, total number of prompts in a study) by the intraclass correlation coefficient (ICC) representing the variation of compliance between- and within-study participants. Eight studies reported the variance in participant compliance, which prevented this review from estimating ICCs separately for each included study. In the 8 studies that reported this information, the range of participant-level standard deviations in compliance was 5.96-29.98%. Accordingly, the sample sizes used to calculate the standard errors were adjusted by ICCs reflecting this range: the meta-analyses were conducted using an ICC calculated assuming lower (SD 5.96%), intermediate (SD 15.00%), and higher (SD 29.98%) values of the ICC, and results were compared in sensitivity analyses. The sensitivity analyses showed that the results were not affected by different ICC values, and only results estimated using the intermediate ICC value are presented here.

The *Q* statistic was computed by summing the squared deviations of each study’s ES from the average pooled ES and was used to test the statistical significance of between-study heterogeneity in compliance rates. The *I*^2^ statistic, which estimates what proportion of the between-study variance is due to actual differences rather than chance, was used to quantify the magnitude of between-study heterogeneity, with values of 25%, 50%, and 75% representing low, medium, and high heterogeneity, respectively [12].

#### Moderator Analyses

The second goal of this meta-analysis was to examine the association between EMA study design characters (average daily prompting frequency and length of monitoring period) and participant compliance rates. Both average daily prompting frequency and length of monitoring were coded based on information described in the reviewed publications. For studies that employed different frequencies for weekends and weekdays, an average daily prompting frequency was calculated by dividing the total number of times participants were prompted by the number of study days.

The associations between study design variables (ie, length of EMA protocols and sampling frequency) and reported compliance were examined using random effects analysis of variance (ANOVAs) with inverse variance weights. Models that examine the association of compliance with (1) study length and (2) daily prompting frequency were estimated separately for studies with participants from nonclinical or clinical populations. The length of study protocol was operationalized as study length ≤1 week, >1 week and ≤2 weeks, and >2 weeks, and the prompting frequency was operationalized as prompting frequency of 2-3 times per day, 4-5 times per day, and ≥6 times per day to ensure that each category included a sufficient amount of studies for the purposes of comparison. We considered testing the interaction term of study length and prompting frequency, but did not conduct this analysis because there would have been no or very few studies in several categories comprising the interaction effect. All meta-analysis procedures were conducted using Comprehensive Meta-Analysis (version 3, Englewood NJ, USA).

## Results

### Study Selection

**Figure 2:**
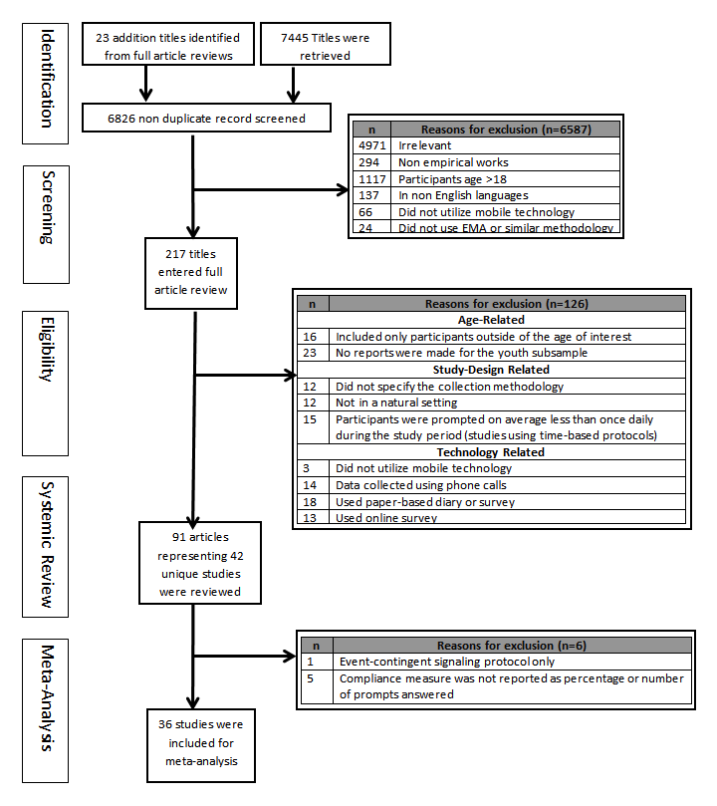
The preferred reporting items for systemic review and meta-analysis (PRISMA) diagram.

A total of 6826 nonduplicate titles were identified. Of these, 6803 were identified using search engines and 23 were identified through cited work from the articles screened. After reviewing abstracts and full-text articles for inclusion and exclusion criteria, 91 empirical articles representing 42 unique studies were included in the qualitative systematic review and 36 studies were included in the meta-analysis portion of the study. The detailed study selection process is outlined in Figure 2, the preferred reporting items for systemic review and meta-analysis (PRISMA) diagram [13].

### Participant Characteristics

Among studies included in the systemic review, the average number of participants in the analytic sample across studies was 98.81 (SD 130.66; range 5-562). Across all studies, the average proportion of female participants was 56.4%, where 3 (7.1%) recruited only female participants. Excluding these 3 studies, the average proportion of female participants was 52.7% (SD 18.7%; range 7.6-86.7%). A majority of the included studies (n=26, 61.9%) recruited only participants from community or nonclinical settings. The 16 studies with clinical populations focused on youths with various health conditions: attention deficient/hyperactivity disorder [14-16] (25%, 4/16), juvenile idiopathic arthritis (JIA) [17-19] (25%, 4/16), asthma (13%, 2/16), type 1 diabetes (T1D) [20,21] (13%, 2/16), high function autism and Asperger’s syndrome (HFASD) [22] (6%, 1/16), concussion patients [23] (6%, 1/16), neurology clinic patients [24] (6%, 1/16), and recovery patients [25] (6%, 1/16). A detailed outline of the study participants can be found in Multimedia Appendix 1.

### Study Characteristics

#### Study Length

The length of EMA protocols ranged from 2 to 42 days (13.27 [SD 9.08]). The average length of monitoring was not statistically different between studies with nonclinical participants (mean 11.4 days [SD 6.9; range 4-30 days]) and clinical participants (mean 16.3 days [SD 11.2; range 2-42 days]; *t*_24.5_=−1.598, *P*=.12).

#### Sampling Strategy

A total of 33 (78.6%) studies utilized only time-based sampling protocols, 1 study (2.4%) used only an event-based sampling protocol, and 8 studies (19.0%) used a combination of both time- and event-based sampling protocols.

Among the 41 studies with a time-based sampling component, prompting schedules included random prompts during predetermined time intervals (n=31, 75.6%; eg, one random prompt for each 2 h interval), prompts at a fixed schedule (n=8, 19.5%; eg, every 30 min during waking hours), prompts at a personalized time (n=1, 2.4%; eg, participant’s own blood glucose check schedule), and one study did not report the prompting scheme. Prompting schemes of which participants were prompted is shown in Multimedia Appendix 2. Studies assessed a wide range of behaviors (eg, current activity, meal consumption, alcohol consumption, self-injury behavior, unprotected sexual behavior, and disease management), subjective experience (pain, mood, stress, appetite, attention, functional limitations, level of enjoyment, and level of control), and contextual information (current physical location, presence of social companion, presence of smoking cue, and food availability).

Among the 9 studies with an event-based protocol, participants in 8 studies were asked to initiate self-report after occurrence of certain thoughts or emotions such as positive feelings [26], negative feelings [26,27], self-injury thoughts [28]), physical symptoms [27], behaviors (ie, drinking [29], smoking [30,31], eating occasion [32], or self-injury behavior [28]), or exposure to smoking or alcohol-related media campaigns [33]. One study [34] automatically sent an EMA prompt approximately 5 min after using a Bluetooth-enabled inhaler. Multimedia Appendix 3 lists the experiences and/or behaviors that the included studies asked their participants to self-report for the event-based protocol.

#### Sampling Frequency

A majority of the included studies (95%, 39/41) prompted their participants 2-9 times during each day of EMA data collection. It was found that 2 (4.9%) studies prompted participants more than 25 times each day and 10 (24.3%) studies reported prompting participants in different frequencies on weekdays versus weekend days. Excluding the two studies that prompted participants more than 25 times each day, the average prompting frequency was 4.2 times per day (range 2-9) for studies with nonclinical participants and 3.6 times per day (range 2-7) for studies with clinical samples.

#### Sampling Devices

EMA data was collected using electronic diaries (n=1, 2.3%), wearable platforms (n=1, 2.3%), iPods (n=2, 4.6%), PDAs (n=10, 23.8%), palmtop computers (n=12, 28.4%), and mobile phones (n=16, 48.1%). A small proportion of studies (n=5, 11.9%) reportedly used participants’ own phone or mobile phones to implement EMA data collection. Four of these studies sent text messages to participants’ own phones or mobile phones for EMA data collection and one allowed participants to choose between using a mobile phone provided by the study and their own smart devices. A small proportion of studies (n=5, 11.9%) used wearable devices in addition to the EMA data collection platform. Devices utilized in addition to the EMA data collection platform included accelerometers [34-37], heart rate monitors [35,36], Bluetooth-enabled inhalers [34], and glucometers [21].

#### Incentive for Participants

A majority of the reviewed studies (n=28, 66.67%) reported the strategy used for incentivizing study participants. Among these studies, most of them (n=26, 92.86%) provided monetary incentive to their participants. Two studies reported using other nonmonetary incentive strategies, for example, raffle [26] and level-up (promotion) in the EMA software platform [18]. Among studies that provided study participants with monetary incentive, study participants were compensated either (1) in a fixed amount (n=16, 57.14%; ranged from US $40 to US $200) or (2) an incremental amount of monetary incentive (n=10, 35.71%). In studies that used the latter approach, participants received a base amount of compensation (ranged from US $20 to US $50) for participation with additional incentive in various rate based on author-specified compliance thresholds. Nonetheless, there is no clear common rationale for determining the level of incentive observed among the reviewed studies. Detailed information about the incentive structure used in these studies can be found in Multimedia Appendix 1.

### Compliance as Reported in the Original Studies

#### Definition of Reported Compliance

Among studies with a time-based sampling protocol component (N=41), the majority (n=36, 87.8%) defined participant compliance as percentage of prompts to which participants responded. Two studies included response latency, or the time difference between a prompt and participant’s response to that particular prompt, as part of the definition of compliance (eg, percentage of prompts responded within 30 min of the first notification [27]). One study provided the percentage of participants who reached a predetermined compliance cutoff (ie, percentage of participants completed 28 entries [28]). The definition of compliance among time-based sampling protocols is listed by study in Multimedia Appendix 2. Among studies that reported compliance in the format of proportion of prompts completed (n=36), the reported compliance rates ranged from 54.6% to 96.21%. Approximately 31% of the reviewed studies (n=13) reportedly excluded participants from the analytic sample because they were considered dropouts or did not meet a minimal compliance threshold.

Among studies with an event-based sampling protocol component (n=9), the majority (n=8, 88.9%) asked participants to initiate self-report. These events to initiate self-reports included occurrence of behaviors [27-32,34], media exposure [33], or subjective experiences [26-28] during the monitoring period (Multimedia Appendix 3). Limited information about compliance was available from the 8 studies with event-based protocols. Among these studies, 6 reported compliance in the format of count of prompts that contained information about the behavior of interest. One (11.1%) study with an event-based protocol asked participants to respond to EMA prompts triggered by events (ie, use of the inhaler) sensed by a Bluetooth-enabled inhaler [34]. In this study, compliance was reported as the proportion of answered prompts triggered by the sensed use of the Bluetooth-enabled inhaler. The compliance rate reported in this study was 47.90%.

#### Reported Correlates of Participant Compliance

Among studies with clinical participants (n=17), 8 examined correlates of compliance and reported no significant association between prompt completion rate and day of the week [18,20], time of day [18,20], age [19,20,22,24,34], gender [18-20,22,34,38], disease status [19,20,22,24,34,38], and technical difficulties [17]. One study reported that prompt completion rate was positively associated with participants’ intelligence quotient (IQ) [22]. Three studies reported declines in completion rates over time reported [17,19,20] and one did not observe such a difference [18]. One study documented reasons for missing assessments included technical issues (“did not hear the notification”) [20] and the timing of the prompt [20].

Reported significant correlates of compliance among studies with nonclinical participants included gender [39-41], ethnicity [36,40], health condition [42], and baseline affect [41,43]. Weekday status [36,44] and participant age [36,40] were reportedly not associated with prompt completion rate. Mixed findings on the correlation of prompting time of the day and completion rate were reported in 4 studies [36,39,44,45]. Declines in participants’ completion rate over the course of the study were reportedly tested in 4 studies [30,31,39,46] and 3 reported a significant completion rate decline [30,39,46]. It was found that 5 studies documented reasons for missed assessments. These reasons included participants’ engagement in competing activities [42,47], device malfunction [36,46,47], not hearing the notification [42], and participant forgetfulness [30,46].

### Average Rates and Moderators of Participant Compliance: Meta-Analysis Results

#### Average Compliance Rate

A total of 36 studies with a time-based EMA protocol were included in the meta-analysis portion of the study. After accounting for the cluster effect of momentary assessments within participants, the average compliance rate across the included studies was 78.26% (95% CI 75.49-80.78%), and the average compliance rate was not associated with the average age or gender proportion of the study participants. The average compliance rates were not statistically different between (1) studies with EMA data collected using one mobile platform (77.44%, 95% CI [73.59-80.88%]) compared with studies using a mobile platform with additional wearable devices (n=5, 73.00%, 95% CI [61.75-81.91%]; *z*=−0.91, *P*=.36); (2) studies using a fixed incentive strategy (n=15, 79.08%, 95% CI [69.08-86.48%]), an incremental incentive strategy (n=10, 72.95%, 95% CI [62.36-81.44%]), and did not report using an incentive strategy (n=10, 80.15%, 95% CI [73.00-85.77%]); and (3) studies with participants from clinical settings (76.92%, 95% CI [70.76-82.11%]) compared with studies with participants from nonclinical settings (79.15%, 95% CI [75.59-82.32%]; *z*=−1.06, *P*=.29). There was substantial between-study variation in compliance rates for both studies with clinical (*I*^2^=48.33%, *Q*_total_=27.09, df=14, *P*=.02) and nonclinical participants (*I*^2^=66.93, *Q*_total_=60.48, df=20, *P*<.001). Thus, the examination of moderators of ESs was warranted.

#### Daily Prompting Frequency as Moderator of Compliance

Daily prompting frequency significantly moderated the compliance rates among clinical (*Q*_between_=9.78, df=2, *P*=.008; *R*^2^=.74) and nonclinical (*Q*_between_=15.13, df=2, *P*<.001; *R*^2^=.44) studies. Among studies with clinical participants, the compliance rates were significantly higher in studies that employed the *most* frequent prompts (6 or more times a day) compared with studies with less frequent sampling of 2-3 times per day (*z*=−2.68, *P*=.007) and 4-5 times per day (*z*=−3.10, *P*.002). Conversely, among studies with only nonclinical participants, the compliance rates were significantly higher in studies that prompted participants *the least* frequently (2-3 times a day), compared with studies with prompting frequencies of 4-5 times (*z*=−3.81, *P*<.001), or 6 or more times per day (*z*=−3.53, *P*<.001; Table 1).

**Table 1:** Prompting frequency by intensity category.

Setting	Prompting frequency (# of prompts per day)	n	Compliance (95% CI)	*R* ^2^_analog_	*Q*_residual_^a^	*I*^2^ (%)^b^
Clinical	2-3 times	11	73.47 (67.45-78.73)_d_	0.74	14.97 (df=12)	19.82
	4-5 times	4	66.94 (53.50-78.09)_e_			
	6+ times	2	89.28 (78.83-94.90)_c_			
Nonclinical	2-3 times	6	91.73 (85.48-95.44)_g_	0.44	38.27 (df=18) *P=*.004	52.96
	4-5 times	13	74.42 (59.37-85.29)_e_			
	6+ times	5	75.00 (59.21-86.12)_f_			

^a^*Q*_residual_: test for residual between-study variance (not explained by the moderator) against zero.

^c^*I*^2^: percentage of the residual variability that is due to heterogeneity rather than sampling error.

^c^*P*=.007, compared to study with a prompting frequency of 2-3 times.

^d^*P*=.007, compared to study with a prompting frequency of 6+ times.

^d^*P*=.002, compared to study with a prompting frequency of 6+ times.

^e^*P*<.001, compared to study with a prompting frequency of 2-3 times.

^f^*P*<.001, compared to study with a prompting frequency of 2-3 times.

^d^*P*<.001, compared to study with a prompting frequency of 6+ times.

**Table 2:** Length of monitoring by week.

Settings	Length of EMA^a^ monitoring	n	Compliance (95% CI)	*R* ^2^_analog_	*Q*_residual_^b^	*I*^2^ (%)^c^
	(number of weeks)					
Clinical	1	6	78.13 (64.37-87.61)	0	26.67 (df=12)	55.01
	2	5	73.46 (53.74-86.84)		*P*=.009	
	3+	6	75.47 (56.86-87.78)			
Nonclinical	1	14	75.81 (70.39-80.52)	0.11	51.43 (df=18)	65
	2	5	76.77 (61.30-87.33)		*P*<.001	
	3	5	83.95 (74.69-90.71)			

^a^EMA: ecological momentary assessment.

^b^Q_residual_: test for residual between-study variance (not explained by the moderator) against zero.

^c^*I*^2^: percentage of the residual variability that is due to heterogeneity rather than sampling error.

#### Study Length as Moderator of Compliance

There were no significant differences in reported compliance between studies that engaged participants in an EMA protocol for 2 and 3 or more weeks compared with studies that engaged participants for 1 week or less, among both studies with participants from clinical (*Q*_between_=0.33, df=2, *P*=.85) and nonclinical (*Q*_between_=2.60, df=18, *P*=.27) settings (Table 2).

## Discussion

### Principal Findings

The aim of this study was to provide an up-to-date review of evidence on youths’ compliance to real-time EMA protocols operated on mobile platforms. Interest in using EMA with mobile technology in youth is growing rapidly, as documented by the sizable number of mobile-EMA studies conducted to capture various aspects of youth’s life. In the reviewed studies, we estimated an average compliance rate of 78.3% across studies using time-based prompting protocols. Although this rate is comparable with the rate of 71% (range 44-96%) observed by Liao et al [9], this study’s estimate is lower than the EMA compliance rate reported in the adult populations [48] and just falls short of the 80% rate recommended by Stone and Shiffman [49]. Considering that close to 30% (n=11) of the reviewed studies reported a compliance rate that is lower than 70%, there is a need to identify factors that may impact youths’ compliance to mobile-EMA protocols in order to facilitate more optimal compliance rates.

### Study Design and Completion Rates in Time-Based Protocols

The study designs varied considerably both in terms of the overall length of EMA monitoring and in terms of the frequency with which youths were prompted to complete momentary assessments per day. This allowed us to examine whether these specific EMA study design factors moderate compliance rates. Our meta-analytic findings provided evidence that the compliance rates are significantly different among studies of different daily frequency of assessments. Importantly, although being significant for both nonclinical and clinical samples, the effect was in opposite directions for clinic and nonclinic participants. Among the 17 studies with participants from *clinical* settings, the two studies with *the highest prompting frequency* had a significantly higher compliance rate of 89.3% compared with studies with a less intense prompting frequency (73.5% for studies with the lowest frequency; 66.9% for studies prompted participant 4-6 times a day). Conversely, among studies with *nonclinical* participants, we estimated an average compliance rate of 91.73% among studies with *the lowest prompting frequency* and was significantly higher than the rate in studies that prompted participants for 4-5 times or 6+ times per day (74.4% and 75.0%, respectively). These results suggest that the association between prompting frequency and compliance differ between participants from nonclinical and clinical settings.

We can only speculate on the potential reasons for this result. One possibility is that studies in nonclinical and clinical settings differ in the content of the questions and how meaningful they are to respondents. Clinical studies commonly tap into medical and disease-related aspects of daily life that may be intrinsically relevant to the young patients. On the other hand, the content of EMA prompts in nonclinical studies may appear less intrinsically relevant to respondents, which may decrease compliance when the assessments are more frequent.

On the other hand, the meta-analytic results indicate that the overall compliance rates were similarly moderate among studies with different lengths (number of weeks) of EMA monitoring in either setting. However, as several reviewed studies with clinical [17,19,20] and nonclinical [30,39,46] participants reported declines in compliance rates over the course of study period, these results suggest that young participants’ compliance to EMA protocols might deteriorate over time. These results emphasize the need for developing a more nuanced understanding of the possible factors and strategies that sustain youths’ motivation to complete EMA prompts over extended periods of time. Several strategies (eg, reward systems, rotating item administration) were utilized as mechanisms for maintaining youths’ motivation to comply with longer EMA protocols. The post hoc analysis results further indicate that the average compliance rates did not differ between studies with a fixed incentive structure and an incremental incentive structure. Although this result suggests that these two incentive strategies may have similar effectiveness in motivating young participants to comply, further investigations on crucial aspects of how these strategies affect participant compliance, for example, the mechanism of which young participants are motivated, is necessary. However, to the authors’ knowledge, there is no published evidence that systemically examined the effectiveness of reward systems or other promising strategies on compliance to EMA among young participants.

### Mobile Technologies Used in Current EMA Protocols

Several studies with a time-based sampling protocol in this review incorporated wearable or deployable devices such as accelerometers [34-37], heart rate monitor [36], GPS trackers [36], and glucometers [21] in their field data collection efforts, *in addition to* the EMA reporting platforms. Since these devices often collect certain behavioral or contextual data passively with minimal inputs required from study participants, incorporating additional wearable devices in EMA data collection may be acceptable to youth without impacting participant compliance. Although providing a detailed review of feasibility and utility of existing mobile technology in youth is beyond the scope of this study, we identified several existing reviews that examined the use of wearable and mobile technology in assessing particular behaviors (eg, physical activity [5,50,51], dietary behavior [5,50,52], and smoking [53]). Considering the suboptimal average compliance rate estimated among mobile-EMA studies using a time-based protocol, combining wearable technologies with EMA data collection may offer a viable alternative to collect some self-reported data to alleviate participant burden. In addition to reducing participant burden, recent developments in human-computer interaction reveal the possibilities of using data captured by mobile phone sensors to identify ideal timing, or the “opportune moments” [54-57], to send prompts in order to minimize interruption or participant’s engagement in competing activities. Several studies with adult participants [55,57,58] have developed algorithms that predict users’ availability to respond and receptivity to be intervened. Although the extent to which data collected using this approach is subject to selection bias and its utility in youth are yet to be determined, utilizing the data passively collected from mobile devices may offer researchers valuable opportunities to understand participant behaviors and improve compliance.

Among the event-based protocols reviewed, only a small proportion of studies (n=1, 12.5%) used a protocol that emits event-based prompts based on objectively measured behavior of interest using wearable devices [34]. Currently, the majority of the mobile-EMA studies operationalize event-based sampling using a participant self-initiated self-report approach. Nonetheless, as self-reported information obtained using this unsolicited approach may subject to systemic under- or over-reporting [59], quantifying participant compliance to this type of protocol using the current common practice (reporting number of event of interest recorded) can be misleading and overly optimistic. Mobile technologies are increasingly sophisticated in capturing objective measurements of various behaviors [5,50-53]. Therefore, incorporating wearable mobile devices in event-based sampling procedure, in parallel with participant self-report, may present researchers the opportunity to capture objectively measured data about participants’ behavior without impacting participant compliance.

### Limitations in the Current Compliance Reporting Practice

Another finding from this review is that there are areas where compliance-related results and procedures were inadequately or inconsistently reported. First, among the time-based protocols, participant compliance was considered to be synonymous with average prompt completion rate (ie, mean percentage of prompt answered). The distribution of compliance rates is often negatively skewed (with the mass of the distribution concentrated at the higher end). If this is the case, the arithmetic mean provides a conservative representation of overall compliance in the sample, and robust measures of central tendency (median or geometric mean) should be reported. In addition, this relatively vague definition of compliance does not account for important information, such as response latency, that could allow for assessment of response patterns or approximation of item cognitive load. Response latency, or the time difference between a prompt and its corresponding response, is especially relevant when assessing experiences that are time-varying and context-dependent (eg, pain, emotion, and hunger). For example, past emotions or experiences like pain are prone to be distorted by events or experiences occurred during the active reconstruction process of recall [60]. As the time-stamp information is automatically collected in modern mobile technologies, incorporating response latency in defining and reporting compliance can provide fine-grain insights to young users’ compliant behavior. Therefore, we recommend that future EMA studies incorporate the time-frame in which the EMA must be completed in order to be considered compliant and report the latency of prompt completion when applicable.

Second, although a number of studies examined correlates of compliance (ie, quantitative assessment of compliance) and participant-reported reasons for noncompliance (qualitative assessment of compliance), results of both quantitative and qualitative assessment of compliance were inconsistently reported across studies, in part, because the data may not have been collected in the study. Obtaining and reporting information about how individual (eg, age and gender), technological (eg, software malfunction, device power depletion, and network connectivity), or time-varying (eg, time of day, environmental factors, and activities) factors relate to compliance and to missing data is important for at least two reasons. For one, identifying these factors is necessary for improvement of compliance in future EMA data collection. For example, by understanding which participant groups need to be specifically targeted (and when they need to be targeted), improved retention strategies can be formulated to address the challenges unique to participants of specific demographic groups and to facilitate overall compliance rates. In addition, without this information one cannot determine if missing data in EMA studies is merely random “noise” or if it is systematically linked to individual or situational characteristics. Systematic noncompliance is clearly a major threat to the validity of conclusions and analytic steps can be taken to attenuate the bias if the attributing factors are known. Therefore, we encourage future EMA studies to report both quantitative and qualitative compliance results.

Third, several studies reported compliance rates only among those in the final analytic sample, after removing participants with low compliance. Many studies provided rationales for excluding low- or noncompliant participants. Nonetheless, the compliance rate reported with these subsamples can be viewed as inflated and would be likely to affect our ability to accurately estimate the average compliance across studies. Therefore, we recommend that future studies report compliance rates before and after removing the participants from analyses to enhance transparency of the analysis process.

### Limitations of This Review

A major strength of this study was that we were able to quantitatively assess the compliance rates for mobile-EMA studies of various health-related behaviors and the association between the reported prompt completion rate and some design factors. Our findings, however, only pertain to two aspects of a real-time EMA protocol (ie, prompting frequency and sampling length) that may affect participants’ compliance [2]. Examples of other important aspects include the quality and complexity of the prompts, effort required to complete each assessment, attractiveness of the interface (ie, developmental appropriateness, and esthetics), and stability of the reporting platforms. To date, however, information on EMA protocol designs is inconsistently reported; therefore, this study was not able to assess the effect of these factors on the reported compliance. In addition, the average compliance rate estimated in this study may be somewhat inflated, as some reviewed studies reportedly excluded low-compliant participants from the analytic datasets used for calculating and reporting compliance. The possibility of overestimating the average compliance rate reflects the methodological limitation of conducting meta-analysis using data extracted from text of published literature. Future research that analyzes data retrieved from each individual study will be able to provide a more precise estimate of participant compliance rate and to further understand how individual participant characteristics affect participant compliance.

### Conclusions and Future Directions

Using mobile technologies as data collection platforms in EMA studies has demonstrated generally moderate, but suboptimal, compliance rates among the youth population. In this review, we have further identified that sampling intensity, a possible proximate of participant burden, might impact compliance of participants from different settings. The study results suggest that youth from nonclinical settings may comply better with mobile-EMA protocol with a lowered daily prompting frequency, whereas youth from clinical settings comply better otherwise. Nonetheless, the nonexperimental nature of this review limits our ability to make further recommendations and highlights the need for experimental studies to investigate the impact of these study design factors on participant compliance. Moreover, this review identified several areas of compliance-related results that are currently inconsistently or inadequately reported among the reviewed studies. This suggests the need for thorough reports of participant compliance, which would potentially advance the current understanding of participants’ compliance to EMA protocols and to aid development of future EMA study designs. Therefore, we suggest that future studies use the proposed reporting guidelines by Liao et al (Multimedia Appendix 2) [9].

We further emphasize the importance for future studies to report results in several areas that have been most inconsistently reported. These areas include (1) the reporting of EMA design features that were used to reduce participant burden or potentially improve data quality (eg, minimizing item “over exposure” by administering items in rotated order); (2) the number of prompts delivered and actually received by the participants, and whether nonresponse was due to technical issues or participant noncompliance; (3) response latency, or the amount of time from prompt signal to prompt answering; (4) distributional characteristics of noncompliance rates (ie, standard deviation and skewness of participant prompt completion rates), participant compliance results based on the full sample to improve the transparency and consistency in reporting prompt response rate; and (5) demographic and time-varying correlates of EMA compliance. Furthermore, we suggest that future studies should incorporate the time-frame information when defining participant compliance. As one of the central promises of EMA the collection of data with a reduced recall bias, providing this information could aid future studies and meta-analytic reviews to determine the effect of latency on data collected, which may further improve the current understanding of participant compliance.

## Appendix

Multimedia Appendix 1 Study characteristics.

Multimedia Appendix 2 Ecological momentary assessment (EMA) study with time-based design.

Multimedia Appendix 3 Ecological momentary assessment (EMA) study with event-based design.

## Abbreviations

ANOVA: analysis of variance
EMA: ecological momentary assessment
ES: effect size
ESM: experience sampling methods
GPS: Global Positioning System
HFASD: high function autism and Asperger’s syndrome
ICC: intraclass correlation coefficient
IQ: intelligence quotient
JIA: juvenile idiopathic arthritis
PDA: personal digital assistant
PRISMA: preferred reporting items for systemic review and meta-analysis
T1D: type 1 diabetes
