# Supplementary material for: Compliance With Mobile Ecological Momentary Assessment Protocols in Children and Adolescents: A Systematic Review and Meta-Analysis
Source: J Med Internet Res. 2017 Apr 26;19(4):e132. doi: 10.2196/jmir.6641 (PMC5425774; doi:10.2196/jmir.6641)
Supplement: Multimedia Appendix 1 [file jmir_v19i4e132_app1.pdf]

Table 1. Study characteristics.

#	Author Year	Sample size	Average age (SD[a]; range)	Gender (%female)	EMA[b] design	Additional mobile device used	Incentive structure
**Nonclinical population**							
1	Dunton 2007	524	14.5 (0.5; NR[c])	NR	Time[d]	Heart rate monitor Accelerometer	Incremental[e]
2	Weinstein 2006	562	8th grader: 13.9 (0.4; NR), 10th grader: 16.0 (0.4; NR)	8th grader: 52, 10th grader: 57.5	Time[d]	NR	Fixed[f]
3	Gwaltney 2007	13	17.4 (0.8; 14-18)	38.5	Mixed[g]	NR	Fixed[f]
4	Reid 2009	18	Male: 15.8 (0.8; 15-17) Female: 15.9 (1.1; 14-17)	68.9	Time[d]	NR	Fixed[f]
5	Nock 2009	30	17.3 (1.9; 12-19)	86.7	Mixed[g]	NR	Fixed[f]
6	Suveg 2010	38	Male: 9 (1.5; 7-12), Female: 9	49.0	Time[d]	NR	Fixed[f]

			(1.9; 7-12)				
7	Dunton 2011	121	11.0 (NR; 9-13)	49.0	Time[d]	Heart rate monitor Accelerometer	Incremental[e]
8	Scharf 2013	20	12.8 (1.6; 11-17)	50.0	Mixed[g]	NR	Fixed[f]
9	Kim 2013	35	13.6 (0.5; NR) (youth subsample)	86.7	Mixed[g]	No	N/A[h]
10	Crooke 2013	41	15.4 (1.0; 14-17)	61.0	Time[d]	NR	Fixed[f]
11	Rusby 2013	82	NR	51.2	Time[d]	NR	Incremental+Other[i]
12	Bickham 2013	91	14 (NR; 13-15)	49.5	Time[d]	NR	Incremental[e]
13	Grenard 2013	158	16.0 (1.0; 14-18)	57.0	Mixed[g]	NR	Fixed[f]
14	Rahdar 2014	45	16.5 (0.8; 15-17) (n=22, youth subsample)	40.1	Time[d]	No	N/A
15	Garcia 2014	24	15 (NR; 14-17)	100.0	Mixed[g]	NR	Other[h]
16	Miranda 2014	65	18.3 (0.9; 15-19) (n=29, youth subsample	55.0	Event[j]	NR	N/A

			)				
17	Ranzenhofer 2014	30	14.9 (1.5; 12-17)	100.0	Time[d]	NR	N/A
18	Johnson 2014	35	12.1 (0.3; 12-13)	63.0	Time[d]	NR	Incremental[e]
19	Hoeppner 2014	202	16.5 (1.2; 14-18)	38.3	Mixed[g]	NR	Incremental[e]
20	Roekel 2014	278	14.2 (0.6; 13-16)	59.0	Time[d]	NR	Fixed[f]
21	Sokolovsky 2014	461	15.7 (0.6; 13.9-17.3)	55.1	Time[d]	NR	Fixed[f]
22	Dunton 2015	166	NR (NR; 8-12)	NR	Time[d]	Accelerometer	Fixed[f]
26	Bjorling 2009	31	16 (NR; 14-18)	100.0	Time[d]	NR	N/A
38	Chin 2016	82	13.9 (0.7; 13-15)	37.5	Time[d]	NR	N/A
40	Russel 2015	151	13.0 (0.9; 11-15)	48.0	Time[d]	NR	Fixed[f]
42	Mason 2015	172	16.2 (1.4; 14-18)	52.5	Time[d]	NR	N/A
**Clinical population**							
23	Whalen 2006	52	ADHD[k] group: 10.6 (1.46; NR), Control group 10.9 (1.12; NR)	35.5	Time[d]	NR	Incremental[e]
24	Lewandowski 2009	6	NR (NR; 14-17)	67.0	Time[d]	NR	N/A
2	Connelly	25	12.3 (2.9;	84.0	Time[d]	NR	N/A

5	2009		8-17)				
27	Helgeson 2009	76	14.5 (1.0; 13-16)	50.0	Time[d]	Blood glucose meter	Fixed[f]
28	Rosen 2012	27	9.29 (1.1; 8-12)	29.6	Time[d]	NR	Incremental[e]
29	Connelly 2012	43	13.2 (2.7; 8-17)	86.0	Time[d]	NR	N/A
30	Rosen 2013	5	NR NR; 8-11)	25.0	Time[d]	NR	N/A
31	Stinson 2013	14	13.2 (2.3; NA)	64.0	Time[d]	NR	Other[i]
32	Borus 2013	36	16.6 (1.5; 14-18)	53.0	Time[d]	NR	Fixed[f]
33	Khor 2014	31	14.5 (1.8; 12.0-19.0)	16.1	Time[d]	NR	Fixed
34	Stinson 2008a	76	13.4 (2.5; 9-18)	77.6	Time[d]	NR	N/A
35	Stinson 2008b	36	12.6 (2.4; 8-17)	66.7	Time[d]	NR	N/A
36	Dennis 2014	29	16.6 (NR; NR)	31.0	Time[d]	NR	Incremental[e]
37	Dunton 2015	20	14.6 (1.7; 12-17)	45.0	Mixed[g]	Accelerometer<br>Propeller sensor	Fixed[f]
39	Rhee 2015	84	15.0 (1.4; 13-17)	61.0	Time[d]	NR	Fixed[f]
41	Bromberg 2015	59	13.3 (2.78; 8-18)	74.6	Time[d]	NR	Incremental[e]

[a]SD: standard deviation.

[b]EMA: ecological momentary assessment.

[c]NR: not reported.

[d]Time-based EMA design studies that prompt study participant to self-report according to a schedule based on time. Time-based designs include prompting participants at random time points, fixed time points, a stratified time block, and so on.

[e]Incremental incentive: Participants were given an incremental amount of monetary incentive based on completion rate of EMA prompts during the monitoring period.

[f]Fixed incentive: Participants were given a fixed amount of monetary incentive after completion of each EMA monitoring period.

[g]Mixed EMA design studies uses a combination of both time-based and event-based schedule, based on the study objectives.

[h]N/A: not applicable.

[i]Other: The study reported using an incentive strategy other than a fixed or an incremental incentive structure. Detailed information about the incentive strategies used in these studies can be found in Supplemental Table S1: Detailed information about the incentive strategies used..

[j]Event-based EMA designs are studies that require participant to initiate self-report or with assessment triggered by the occurrence of a predefined event of interest.

[k]ADHD: attention deficient/hyperactivity disorder.
