# Supplementary material for: Compliance With Mobile Ecological Momentary Assessment Protocols in Children and Adolescents: A Systematic Review and Meta-Analysis
Source: J Med Internet Res. 2017 Apr 26;19(4):e132. doi: 10.2196/jmir.6641 (PMC5425774; doi:10.2196/jmir.6641)
Supplement: Multimedia Appendix 2 [file jmir_v19i4e132_app2.pdf]

Table 2. Ecological momentary assessment (EMA) study with time-based design.

#	Author Year	Prompting schedule	Length of study (days)	# of prompt per day	Reported compliance mean (SD[a]; range)	Compliance definition
27	Helgeson 2009	Every 2 h during wake time on weekend days	2	6-8	7 (NR[b]; 2-9) entries per day	# of prompts answered[c]
42	Mason 2015	Random time: After 4:00 pm to evening	4	3	NR	N/A[d]
7	Dunton 2011	Random time: 3 on weekday 7 on weekend	4	3-7	80.0% (NR; 7-100%)	% of prompts answered
1	Dunton 2007	Every 30 min (SD 10) during waking hours	4	25+[e]	83.0% (9.4; NR)	% of prompts answered
24	Lewandowski 2009	Sampling Prompts at(1) 9:00-10:00, (2) 11:00-12:00, (3) 14:00-15:00, (4) 17:00-18:00, and (5) 20:00-21:00	5	5	93.3% (NR; NR)	% of prompts answered overall[f]
20	Roekel 2014	Random in each 90-min interval	6	9	37 (11.1; NR) entries	# of prompts answered individually[g]
39	Rhee 2015	One random prompt each at: (1) 6:00-12:00	7	2	NR	N/A

		(2) 18:00-0:00				
3<br>8	Chin<br>2016	Once random prompt each at:<br>(1) morning<br>(2) afternoon	7	2	96.2% (NR; NR)	% of prompts responded individually
9	Kim<br>2013	One random prompt each at:<br>(1) 12:50<br>(2) 16:30<br>(3) 20:00	7	3	95.5% (6.0; NR)	% of prompts answered individually within 30 min from the first signal
4	Reid<br>2009	One random prompt each at:<br>(1) 8:00-11:00<br>(2) 11:00-15:00<br>(3) 15:00-20:00<br>(4) 20:00-22:00	7	4	76.0% (NR; NR-28)	% of prompts answered
1<br>8	Johnson<br>2014	Fixed time:<br>(1) 7:30<br>(2) 11:20am or 11:40am<br>(3) 4:15<br>(4) 7:30	7	4	80.2% (14.2; 40.9-100%)	% of prompts answered individually
6	Suveg<br>2010	Random time:<br>4 times between 4pm and 8pm	7	4	60.0% (NR; NR)	% of prompts answered
2<br>2	Dunton<br>2015	Random time:<br>3 on weekday	7	3-7	69.0% (NR; 25-100%)	% of prompts answered individually

		7 on weekend				
37	Dunton 2015	Once every 2 h<br>(1) 15:00-21:00 (weekday)<br>(2) 7:00-21:00 (weekend)	7	3-7	54.6% (NR; NR)	% of prompts answered
11	Rusby 2013	Every 90-120 min between:<br>Monday to Thursday: 15:30-21:30<br>Friday: 15:30-23:00<br>Saturday: 11:30-23:00 Sunday: 11:30-21:30	7	4-6	75.0% (NR; NR)	% of prompts answered individually by waves
21	Sokolovsky 2014	Random time	7	5-7	68.1% (16.9%; NR)	% of prompts answered individually
2	Weinstein 2006	Random time	7	5-7	85.0% (14.0%; NR)	% of prompts answered individually
23	Whalen 2006	Each 30 min (SD 5) during nonschool hours	7	25+[e]	NR (NR; 71-100%)<br>ADHD[h]: 89.0% (NR; NR)<br>Control:90.0% (NR; NR)	% of prompts answered individually
13	Grenard 2013	Once each<br>(1) 9:00-12:00 (weekend)<br>(2) 12:00-15:00 (weekend)<br>(3) 15:00-18:00	9	3-5	71.0% (NR; NR)	% of prompts answered

		(4) 18:00-21:00				
31	Stinson 2013	Once each:<br>(1) morning<br>(2) afternoon<br>(3) evening	14	2	81.0% (22%; NR)	% of prompts answered individually
8	Scharf 2013	2 random prompt between 10:00 and 22:00	14	2	82.0% (NR; NR)	% of prompts answered individually within 2 min
5	Nock 2009	Once each at<br>(1) mid-day<br>(2) end-of-day	14	2	83.3%	% of participants completed at least 28 entries
14	Rahdar 2014	NR	14	3	NR	N/A
25	Connelly 2009	Once each at:<br>(1) before school<br>(2) after school<br>(3) evening	14	3	84.0% (NR; NR)	% of prompts answered individually
34	Stinson 2008[e]	Once each at:<br>(1) morning<br>(2) afternoon<br>(3) evening	14	3	78.0% (NR; NR)	% of prompts answered
17	Ranzenhofer 2014	Once each during:<br>(1) 11:10 (weekend)<br>(2) 13:50 (weekend)<br>(3) 16:30<br>(4) 19:10	14	3-5	69.4% (NR; 38-90%)	% of prompts answered individually

		(5) 23:50				
15	Garcia 2014	Weekdays:<br>(1) 06:30<br>(2) 14:30<br>(3) 18:00<br>(4) 21:00<br>Weekends:<br>(1) 10:30<br>(2) 15:00<br>(3) 18:00<br>(4) 21:30	14	4	79.6% (NR; 70.5-88.7)	% of prompts answered individually
33	Khor 2014	Once each during:<br>(1) 07:30-08:30<br>(2) 17:00-18:20<br>(3) 18:20-19:40<br>(4) 19:40-21:00	14	4	61.8% (30.0; NR)	% of prompts answered
32	Borus 2013	Personalized time:<br>4 times, according to participants' blood glucose check time	14	4	63.0% (NR; 0-97%)	% of prompt answered (median)
12	Bickham 2013	Random intervals	14	4-7	66.0% (NR; NR)	% of prompts answered
10	Crooke 2013	Once during:<br>(1) 08:00-11:00<br>(2) 11:00-15:00<br>(3) 15:00-20:00	20	4	NR	N/A

		(4) 20:00-22:00				
35	Stinson 2008[i]	Once each at: (1) morning (2) afternoon (3) evening	21	3	73.0% (NR; NR)	% of prompts answered
3	Gwaltney 2007	Once every 3.5-h interval between 8:00 and 22:00	21	4	75.0% (NR; 53-94%)	% of prompts answered
19	Hoeppner 2014	Once every 3.5-h interval between 8:00 and 22:00	21	4	88.4% (6.6%; NR)	% of prompts answered individually
26	Bjorling 2009	7 random time prompts during wake time	21	7	72.0% (NR; 30-90%)	% of prompts answered individually
28	Rosen 2012	Once each at: (1) before school (2) after school (3) evening	28	3	77.0% (NR; 1.5; 46-NR)	% of prompts answered individually
29	Connelly 2012	Once each at: (1) after school (2) dinner time (3) before bed	28	3	2.1 (0.46; NR)	# of prompts answered individually per day[j]
30	Rosen 2013	Once each at: (1) before school (2) after school (3) evening	28	3	84.4% (NR; NR)	% of prompts answered individually
41	Bromberg 2015	Once each at:	28	3	56.2% (18.4; 25-91)	# of diaries completed[k]

		(1) morning<br>(2) afternoon<br>(3) evening				
40	Russel 2015	Once each during:<br>(1) 07:00-10:00<br>(2) 14:00-17:00<br>(3) 17:00-24:00	30	3	92.0% (NR; NR)	% of prompts answered
36	Dennis 2014	Once every block of time that ranged from 90 to 360 min	42	6	89.0% (NR; NR)	% of prompts answered

[a]SD: standard deviation.

[b]NR: not reported.

[c]Converted to 77% (NR; 11.1-100.0%) for meta-analysis.

[d]N/A: not applicable.

[e]Other frequency: Participants were prompted every 30 min during their waking hours, yielding an average 25-30 of recording opportunities per day.

[f]Only among concussed participants.

[g]Converted to 68.52% (NR; NR) for meta-analysis, based on a maximum of 54 prompts per day.

[h]ADHD: attention deficient/hyperactivity disorder.

[i]Two separate studies were reported in the Stinson 2008 paper.

[j]Converted to 68.67% (NR; NR; 41-100%) for meta-analysis, based on a maximum of 3 prompts per day.
