# Supplementary material for: Compliance With Mobile Ecological Momentary Assessment Protocols in Children and Adolescents: A Systematic Review and Meta-Analysis
Source: J Med Internet Res. 2017 Apr 26;19(4):e132. doi: 10.2196/jmir.6641 (PMC5425774; doi:10.2196/jmir.6641)
Supplement: Multimedia Appendix 3 [file jmir_v19i4e132_app3.pdf]

Table 3. Ecological momentary assessment (EMA) study with event-based design.

#	Author Year	Condition to reporting	Event-based compliance	Definition of event-based compliance
16	Miranda 2014	Immediately before and after the first 3 drinking events	26 participants reported at least one drinking episode (2.1 [SD 1.0])<br>94% of the reports were recorded after consumed between 0.67 and 1.33 standard drinks	Classification of compliance:<br>if the successive difference in eBAC[a] across end-drink report is ascending
9	Kim 2013	Report when:<br>1. waking or sleeping<br>2. episode of feeling bad or physical symptoms	1.18 (0.88; NR[b]; NR)	# of event-based EMA[c] recording per day
13	Grenard 2013	eating event: participants were asked to record food or drink consumed within 15 min after consumption	2124 eating occasion were reported as "event-based reporting," an average of 0.94 per participant day)<br>95% evening report completed	N/A[d]
5	Nock 2009	Self-injury thoughts and behavior (multiple choices) for both time-based and event-based	1227 entries (mean 40.9 per person [SD[e] 21.2; range 5-108) that described 1262 episodes of self-destructive<br>thoughts and behaviors	# of entries of event of interest
8	Scharf 2013	Anytime when they are exposed to alcohol- or smoking-related media	8.5 (5.82; NR; NR) (alcohol-related media)<br>4.25 (3.67; NR; NR) (smoking-related media)	Occasion of alcohol- or smoking-related media exposure
3	Gwaltney 2007	Event-based (prequit) every time participant smoked a cig	12/12 participants entered at least one smoking event after	N/A

		Event-based (postquit) every time participant smoked a cig	"I've Quit," or lapsed<br>13.0 (SD 12.4) (2-41) lapses were recorded<br>12/12 participants recorded at least one temptation during the post quit monitoring<br>5.2 (SD 2.7) (1-10) temptations were recorded	
19	Hoeppner 2014	Event-based (prequit) every time participant smoked a cig<br>Event-based (postquit) every time participant smoked a cig	N/A	N/A
15	Garcia 2014	Participants were instructed to initiate open-ended report as positive or negative event happened, in addition to being prompted 4 times a day as described above.	71.21 (NR; 61.97-80.45; NR)	% of prompts responded
37	Dunton 2015	automated prompts approx 5 min after usage of Bluetooth-enabled Propeller sensor	47.90%	% of event-based signal responded

[a]eBAC: estimated blood alcohol calculator.

[b]NR: not reported.

[c]EMA: ecological momentary assessment.

[d]N/A: not applicable.

[e]SD: standard deviation.
